# NAFLD and Cardiovascular Diseases: Epidemiological, Mechanistic and Therapeutic Considerations

**DOI:** 10.3390/jcm10030467

**Published:** 2021-01-26

**Authors:** David Niederseer, Bernhard Wernly, Elmar Aigner, Felix Stickel, Christian Datz

**Affiliations:** 1 Department of Cardiology, University Heart Center Zurich, University Hospital Zurich, University of Zurich, 8091 Zurich, Switzerland; david.niederseer@usz.ch; 2 Department of Anaesthesiology, Perioperative Medicine and Intensive Care Medicine, Paracelsus Medical University Salzburg, 5020 Salzburg, Austria; bernhard@wernly.net; 3 Center for Public Health and Healthcare Research, Paracelsus Medical University Salzburg, 5020 Salzburg, Austria; 4 Department of Cardiology, Paracelsus Medical University Salzburg, 5020 Salzburg, Austria; 5 First Department of Medicine, Paracelsus Medical University, 5020 Salzburg, Austria; e.aigner@salk.at; 6 Department of Gastroenterology, University Hospital Zurich, University of Zurich, 8091 Zurich, Switzerland; felix.stickel@uzh.ch; 7 Department of Internal Medicine, General Hospital Oberndorf, Teaching Hospital of the Paracelsus Medical University, 5110 Oberndorf, Austria

**Keywords:** diabetes, metabolic syndrome, lifestyle, atherosclerosis, NASH, liver, exercise, nutrition

## Abstract

Overwhelming evidence suggests an association of cardiovascular disease (CVD) with non-alcoholic fatty liver disease (NAFLD); however, the underlying mechanisms remain largely speculative. It is, however, likely that common mechanisms contribute to the development of CVD and NAFLD, with lifestyle factors such as smoking, sedentary lifestyle with poor nutrition habits and physical inactivity being major candidates. These behavioral factors, on a predisposing genetic background, trigger changes in gut microbiota, inflammation, dyslipidemia and oxidative stress, leading to metabolic syndrome, diabetes and obesity as well as atherosclerosis. Treatment options to counteract both the progression and development of CVD and NAFLD include lifestyle interventions, optimal medical therapy of comorbid conditions and, as final possibility, bariatric surgery. As no causal pharmacotherapy of NAFLD is available, further research is urgently needed to address the unmet need of a growing population with NAFLD and CVD.

## 1. Introduction

During the last decade, strong evidence has demonstrated a significant interplay and multifaceted relationship between non-alcoholic fatty liver disease (NAFLD) and cardiovascular disease (CVD). Pathophysiological mechanisms associating NAFLD with CVD are incompletely understood, and the current literature on the role of NAFLD as an independent risk factor for CVD and excess CV mortality in NAFLD patients has yielded conflicting results and should therefore be interpreted with caution [1,2,3,4,5]. Nevertheless, NAFLD should be regarded as a systemic metabolic disease affecting extrahepatic tissues throughout the body via complex mechanisms, including diet-induced alterations of the gastrointestinal tract, adipose tissue inflammation and lipotoxicity. Here, we aim to summarize the available data on epidemiological links of NAFLD to CVD and the possible mechanisms as well as current and future treatment options.

## 2. Epidemiological Data Linking NAFLD to Cardiovascular Disease (CVD)

**Figure 1:**
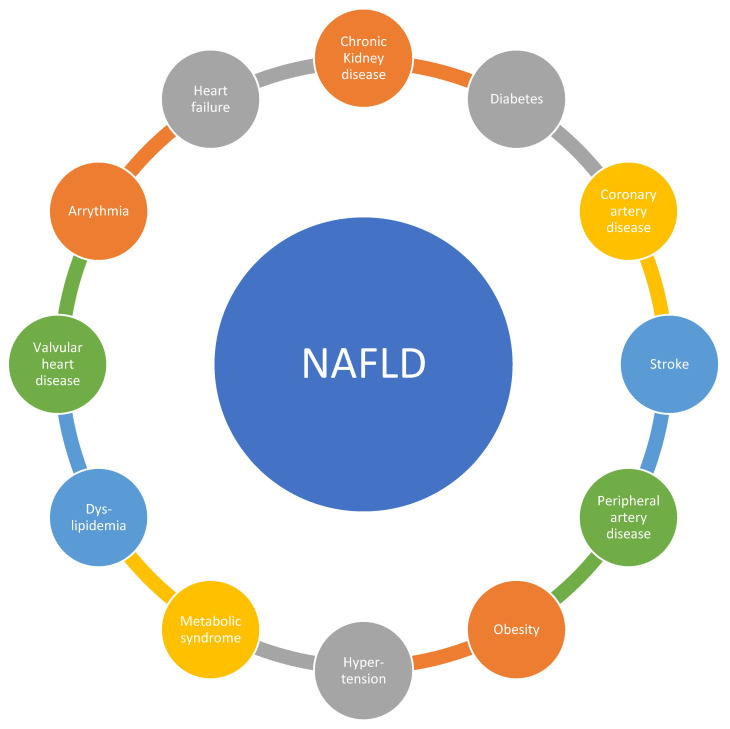
Substantial epidemiological data that link non-alcoholic fatty liver disease (NAFLD) to cardiovascular disease (CVD).

There are substantial epidemiological data that link NAFLD to CVD. We discuss vascular and metabolic disease but also other CV conditions (Figure 1).

### 2.1. Vascular Disease: Atherosclerosis Including Stroke, Peripheral Artery Disease, and Coronary Artery Disease

Although the understanding, prevention, and therapy of non-communicable diseases (NCDs) have improved during recent decades, NCDs constitute the primary cause (around 70%) of death worldwide [6]. Among NCDs, cardiovascular disease (CVD) is responsible for about half of the observed mortality [6]. Besides mortality, CVD leads to high morbidity and costs in Western societies [7,8,9]. CVD can be considered a phenotype with multiple interlapping mechanisms, which include arterial hypertension, obesity, diabetes, socioeconomic factors, and behavioral factors such as physical inactivity and poor nutrition as well as genetic factors, age, and sex [10,11].

NAFLD and CVD share several risk factors. Like CVD, NAFLD is highly prevalent in the total population of developed countries with a prevalence between 20% and 30% [12]. However, in obese patients and patients with diabetes, these proportions increase to up to 90% [13]. Both NAFLD and CVD are more common in men than women, although NAFLD progresses faster in women [13]. More than half of the patients diagnosed with NAFLD suffer from hyperlipidemia, an established independent risk factor for cardiovascular disease [10,11,14]. Furthermore, low-grade systemic inflammation was reported in both NAFLD and CVD [15]. Some experts consider NAFLD as the hepatic manifestation of metabolic syndrome, insulin resistance, and type 2 diabetes mellitus (T2DM) [16].

As both NAFLD and CVD are diseases with high prevalence, sharing several risk factors, it comes as no surprise that epidemiological data supporting NAFLD and CVD coincidence are abundant [1]. Although traditionally considered a hepatic disease, a growing body of evidence suggests that cardiovascular disease in patients with NAFLD determines the outcome rather than liver disease progression [17,18,19].

Observational studies suggest a link between NAFLD diagnosis and an increased risk for CVD and cardiovascular events, including death [4,20,21]. Markers of endothelial dysfunction such as increased intima media thickness and lower flow-mediated dilatation are more common in NAFLD patients [15,22,23]. The risk of stroke was higher in subjects with NAFLD than the general population [15]. Elevated brachial ankle pulse wave velocity and increased arterial stiffness are other surrogate parameters for subclinical atherosclerosis described in NAFLD [24,25].

NAFLD patients evidence higher cardiovascular risk according to the Framingham risk score [26]. Several meta-analyses support the association of NAFLD with increased risk for CVD, although the link between NAFLD and CVD is less robust than between NAFLD and metabolic syndrome [3,16,27]. While some studies have found associations between NAFLD surrogates and CVD, many of these analyses are limited by insufficient adjustment for established cardiovascular risk factors [28,29]. The crucial question of whether NAFLD is a risk factor for CVD independent of metabolic syndrome is, therefore, not yet completely clear. However, since the inclusion of NAFLD in risk calculations for CVD has so far brought no added value compared to models that only consider classical metabolic risk factors, it is, based on the available evidence, currently more likely that NAFLD has no independent impact on cardiovascular outcome beyond their common metabolic background [30,31,32].

### 2.2. Metabolic Disease: Metabolic Syndrome, Dyslipidemia, Diabetes, and Obesity

NAFLD is closely embedded into metabolic syndrome (MetS), which describes the typical common occurrence of established cardiovascular risk factors such as overweight/obesity, dyslipidemia, and disordered glucose homeostasis, as indicated by insulin resistance (IR) or T2DM and arterial hypertension [33]. The prevalence of NAFLD increases with the number of manifestations of metabolic syndrome components [34]. Most NAFLD subjects are obese, and the prevalence in the general population increases with progressive degrees of obesity, approximating almost 95% in subjects undergoing metabolic surgery [34,35]. Likewise, it approaches 60–70% in subjects with T2DM [36]. However, increasing BMI as an indicator of excess adipose tissue appears not to be the sole determining or sufficient factor for NAFLD development as 10% of lean subjects also have NAFLD, and a small proportion of even morbidly obese subjects maintain a healthy liver [37,38,39]. The finding of NAFLD in the absence of excess body weight is commonly referred to as “lean NAFLD” [40]. Further studies have elucidated the central relevance of adipose tissue distribution to NAFLD development as particularly in lean subjects or subjects with a low degree of overweight, substantial visceral fat accumulation is present and directly linked to the presence of a fatty liver [41,42], A meta-analysis by Young et al. demonstrated that lean NAFLD versus healthy subjects or lean controls had higher odds for abnormalities on their metabolic profile, including metabolic syndrome and its components, glycemic dysregulation, renal and liver function, and patatin-like phospholipase domain-containing protein 3 (PNPLA3) G allele, and their inflammatory profile, including uric acid and C-reactive protein [43]. Moreover, Golabi et al. have already demonstrated that lean NAFLD subjects have a higher risk of cardiovascular mortality and overall mortality than normal subjects [44]. In our own experience, lean NAFLD is associated with metabolic syndrome and increased cardiovascular risk as assessed by the Framingham Risk Score (Semmler et al., under review). Hence, limited healthy expandability of subcutaneous adipose tissue appears to represent a pivotal mechanism that triggers fat deposition in ectopic regions such as visceral fat depots and the liver. IR is the unifying underlying mechanism of metabolic complications such as CVD and NAFLD [45]. The importance of IR as a driver for metabolic complications is exemplified by the increasing morbidity and mortality risk with the progression of IR to T2DM [46,47]. This increase in morbidity and mortality in T2DM is true for liver-related complications (i.e., end-stage liver disease and hepatocellular carcinoma (HCC)) as well as for CVD (i.e., heart failure and ischemic events) [48,49]. Thus, the close interrelations are well confirmed epidemiologically between the various manifestations of insulin resistance or MetS on one side and NAFLD on the other; however, these frequently reciprocal relationships make it difficult to ascertain a specific contribution of NAFLD to CVD, as the biological co-evolution allows interpretation as shared risk factors, mutual augmentation, and confounding and may even be due to reverse causation, as genetic analyses indicate [50] (e.g., the possibility of increased prevalence of NAFLD in subjects with CVD due to less physical activity).

### 2.3. Other Cardiovascular Disease Manifestations

Several studies have reported epidemiological associations of NAFLD and valvular heart disease besides vascular and metabolic disease, i.e., mitral annulus calcification and aortic sclerosis. Subsequently, mitral regurgitation and aortic stenosis might develop, which increase the risk of heart failure on the basis of left ventricular dilation (in mitral regurgitation) and left ventricular hypertrophy (in aortic stenosis). Furthermore, arrhythmias have been reported in NAFLD, mainly atrial fibrillation (due to left atrial enlargement secondary to increased filling pressures and mitral regurgitation), increased QTc interval (corrected QT interval on electrocardiogram), and degenerative cardiac conduction disease (mainly first-degree atrioventricular block and left anterior hemiblock) [51,52]. It was recently suggested that NAFLD and non-alcoholic steatohepatitis (NASH) are associated with expansion of epicardial adipose tissue and the release of pro-inflammatory adipocytokines that cause microcirculatory dysfunction and fibrosis of the adjoining myocardium, resulting in atrial fibrillation as well as heart failure with a preserved ejection fraction (HFpEF). Inflammatory changes in the left atrium lead to electroanatomical remodeling; thus, NAFLD and NASH markedly increase the risk of atrial fibrillation. Simultaneously, patients with NAFLD or NASH commonly show diastolic dysfunction or latent HFpEF [53].

Finally, the prevalence of chronic kidney disease (CKD, estimated glomerular filtration rate (eGFR) < 60 mL/min/1.73 m^2^, abnormal albuminuria, or overt proteinuria) is markedly increased in patients with NAFLD. The prevalence of CKD ranged from approximately 20% to 55% in patients with NAFLD as compared to 5% to 30% in patients without NAFLD, and the significant association of CKD and NAFLD mostly persisted after adjusting for confounding factors such as hypertension and T2DM [54,55].

## 3. Possible Mechanisms That Might Explain the Association between NAFLD and CVD

**Figure 2:**
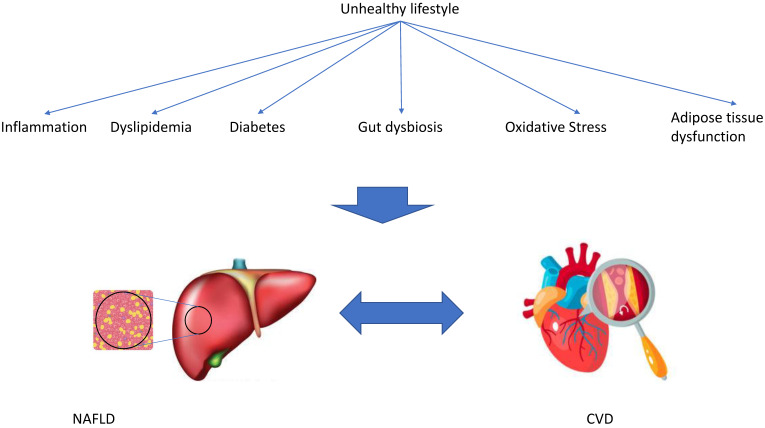
Several possible mechanisms have been proposed that might explain the association between non-alcoholic fatty liver disease (NAFLD) and cardiovascular disease (CVD). All of these possible mechanisms are associated with lifestyle factors.

Several possible mechanisms have been proposed that might explain the association between NAFLD and CVD, including inflammation and oxidative stress, gut microbiota, and dyslipidemia. All of these possible mechanisms are associated with lifestyle factors (Figure 2).

### 3.1. Inflammation and Oxidative Stress

Metabolic inflammation may be regarded as a sterile condition and is a crucial component in the pathogenesis of obesity, T2DM, CVD, and NAFLD, accompanied by increased levels of acute-phase proteins [56,57]. Metabolic inflammation is characterized by a systemic, low-grade inflammatory process in response to several non-infectious factors such as unhealthy dietary habits, especially a high-fat diet [58,59]. This so-called lipotoxicity activates inflammatory pathways and components of the immune system and has been observed in the liver, in arterial vessels, adipose tissue, muscle, pancreas, and the central nervous system. Various compartments such as the liver, the gastrointestinal tract, and adipose tissue are significant sources of pro-inflammatory drivers, including tumor necrosis factor (TNF), interleukin-6 (IL-6), interleukin-1β (IL-1β), C-reactive protein, fibrinogen, and fetuin A [60,61].

Dysregulation of lipid and glucose metabolism and oxidative processes play a key role in the pathogenesis of metabolic dysregulation in obesity, dysglycemia, NAFLD, and atherosclerosis [62,63,64,65,66]. Perturbation of endoplasmic reticulum (ER) homeostasis and oxidative stress have been shown to impact metabolic inflammation by inducing endothelial dysfunction, predisposing patients with NAFLD to develop CV events [67,68]. Persistent ER stress and mitochondrial dysfunction contribute to pathophysiological changes and play an important role in the progression from NALFD to NASH. Calcium ion (Ca^2+^), a critical and versatile intracellular secondary messenger, is involved in various cellular processes. The ER is known to be the most important intracellular Ca^2+^ store. The abnormal release of ER Ca^2+^ not only induces ER stress and mitochondrial dysfunction but also exacerbates hepatic cell lipotoxicity. The sarcoplasmic/endoplasmic reticulum calcium ATPase (SERCA) pump, the main regulator of intracellular Ca^2+^, actively re-accumulates released Ca^2+^ back into the ER and therefore maintains Ca^2+^ homeostasis. SERCA activity is reduced in NALFD, while enhanced SERCA activity alleviates ER stress and apoptosis. Recent studies showed that the homeostasis of Ca^2+^ is closely related to the development of NALFD to NASH [69].

Expanded adipose tissue is a significant source of pro-inflammatory mediators since an estimated excess of 20–30 million macrophages accumulate with each kilogram of excess fat in humans, and adipose tissue hypoxia has a negative impact on the production of anti-inflammatory cytokines [70,71]. Dysfunctional adipocytes act as antigen-presenting cells and express pro-inflammatory cytokines such as TNF-alpha, IL-6, Il-1β, MCP4/CCL13, RANTES/CCL5, and MCP1/CCL2 [72,73,74,75]. The C-C motif Chemokine ligand-2 (CCL2) has been shown to be a key mediator of a cross-talk among adipocytes, macrophages, and endothelial cells potentially aggravating the inflammatory state, resulting in increased expression of pro-inflammatory cytokines, chemokines, adipokines, and angiogenic factors [76]. Genetic deficiency of chemokine receptor 2 (CCR2) and pharmacological antagonism of CCR2 in mice lowered macrophage content and the inflammatory profile of adipose tissue and ameliorated hepatic steatosis. Interestingly, the degree of liver inflammation in patients with NASH is correlated with increased expression of genes that regulate inflammation in adipose tissues [77]. Collectively, adipose tissue inflammation contributes to systemic inflammation, thereby promoting IR and CVD and potentially leading to deterioration of NAFLD [78,79].

Liver inflammation in NAFLD may be regarded as a multidirectional process where inflammatory stimuli may arise from extrahepatic tissues such as adipose tissue and the gut and inside the liver [80]. The role of inflammation in the progression from NAFLD to NASH, cirrhosis, and HCC is incompletely understood and has been somewhat disregarded in recent years as long-term outcomes of patients with NAFLD, including cardiovascular mortality, have been correlated with liver fibrosis [81]. It is well documented that liver inflammation mostly precedes or drives fibrosis [80]. Steatohepatitis may be a highly dynamic process of chronic relapsing or intermittent nature characterized by progression or “spontaneous” fibrosis regression. NAFLD, as a chronic inflammatory condition, may contribute via complex pathways to the systemic metabolic inflammation associated with atherosclerosis and impaired cardiac function [60,82,83]. Hepatic necro-inflammation is likely to be an independent proatherogenic mechanism that could possibly lead to an increased risk of CVD in patients with NASH compared with patients lacking histological features of liver inflammation [84,85]. In contrast, therapeutic antagonism of pro-inflammatory cytokines leads to improvement of hepatic steatosis, liver inflammation, and fibrosis [86,87]. Based on the insights gained from the significance of systemic metabolic dysregulation and its role in disease progression, several consensus statements and experts have recently proposed to rename NAFLD as metabolic-associated fatty liver disease (MAFLD) [88,89,90,91,92]. In order to underpin the importance of metabolic inflammation in the pathophysiology of this disease, highly sensitive C-reactive protein levels have been included as one crucial metabolic risk factor for the disease definition [89,93]. Although this new definition is a big step forward in the nomenclature, several authors have already raised their concerns about this definition; Younossi et al. [94] highlight that the heterogenous nature of NAFLD might not be fully covered by the new MAFLD criteria. In a study by Lin et al. (2020) [95], 620 of 4347 NAFLD patients did not meet the MAFLD criteria (14.3%) [96]. Although metabolic parameters were lower or less frequent in these patients, some had severe steatosis and/or advanced fibrosis (assessed non-invasively) [97].

However, it is unclear whether the new definition can identify patients at increased risk for cardiovascular events or liver-related morbidity and how patients who do not meet these criteria should be managed.

Similarly to differences in the metabolic characterization, differences in the prevalence of MAFLD might be predominantly explained by the use of a more sensitive tool to diagnose NAFLD (i.e., liver stiffness measurement, including controlled attenuation parameter), which probably allowed for identifying patients either at an earlier stage of disease or with a milder phenotype. Thus, associated metabolic disturbances are lower.

To sum up, although the new nomenclature of MAFLD can facilitate diagnosis and patient education, the utility of this new definition still needs to be confirmed.

A conceptual framework of proposed molecular and cellular mechanisms contributing to the metabolic burden of NAFLD on atherosclerosis has recently been published elsewhere [98].

In summary, future studies should be aiming at unraveling the role of hepatic inflammation as a major contributor to systemic metabolic inflammation in the pathogenesis of atherosclerosis and other cardiovascular impairments.

### 3.2. Gut Microbiota

A plethora of microorganisms such as bacteria, archaea, fungi, phages, and viruses colonize the gastrointestinal tract. The microbial community has a strong mutualistic relationship with its host and plays a vital role in digestion, immunity, and human metabolism [99,100]. Tremendous efforts have been made over the last years in elucidating the role of the gut microbiota in fueling metabolic inflammation and unraveling the impact of intestinal microbial composition on the development and disease progression of NAFLD and CVD [101,102,103,104]. The vast majority of intestinal microbiome-related studies assess compositional changes in the fecal microbial community, leading to overwhelming associative data, but are often limited when it comes to causality. Most of these studies were performed in animals; research in humans has only just started to describe microbiome-associated alterations in metabolic diseases. Several studies identified microbiome signatures linked to obesity and metabolic diseases such as T2DM, NAFLD, and cardiometabolic disorders [105,106,107,108,109,110]. A potential link between a specifically dysregulated gut microbiome in NAFLD, NASH, NAFLD-cirrhosis, and HCC discriminating these patients from healthy individuals has been recently presented [102,111,112,113,114,115]). Interestingly, significant changes in the gut microbiome signature have been observed in lean NAFLD [116,117]. The most compelling evidence for a specific intestinal microbiome signature in fibrotic NASH and NAFLD-cirrhosis comes from two recently published studies [102,113]. In their first study, the authors identified an association of advanced fibrosis with abundance of *Proteobacteria* and *Escherichia coli* and a decrease in *Firmicutes* such as *Faecalibacterium prausnitzii*. In a second study, they described a core gut microbiome signature that can identify patients with cirrhosis across different geographical backgrounds, independent of the effects of environmental factors and host genetics. Interestingly, several *Veillonella* species were commonly altered in the cirrhosis groups. In addition, profound changes in the intestinal virome signature have recently been identified in patients with NAFLD [118]. These findings have enormous potential to develop a diagnostic panel that could serve as a non-invasive biomarker for liver disease diagnosis and disease progression.

The close relationship between the gut, the liver, and (visceral) adipose tissue (AT) anatomically and functionally has led to intriguing concepts termed “gut–liver axis” and “AT-liver axis”, as excellently summarized recently [79]. These concepts demonstrate that any change in the gut microbial community not only triggers intestinal disorders but also impacts distant organs and causes associated diseases. It is obvious that gut microbial variations and the development of metabolic diseases such as NAFLD and CVD are tightly interrelated via shared common pathways, including metabolic inflammation [119].

Obesity and related disorders are characterized by profound alterations of the intestinal microbiota composition and functional capacities, raising the concept of intestinal dysbiosis, which, in conjunction with a defective and impaired mucosal barrier, may lead to the translocation of bacteria, bacterial metabolites, bile acids, lipopolysaccharides (LPS), endogenously generated ethanol, and endotoxins into the blood stream, which may result in systemic low-grade inflammation [101,120,121,122,123].

An imbalance of the intestinal microbial composition impacts hepatic fat accumulation via decreased fasting-induced adipocyte factor (FIAF) and increased energy delivery in the form of short-chain fatty acids (SCFAs) produced by the gut microbiota [107,124].

Dietary components such as carnitine, choline, and phosphatidylcholine are converted by gut bacteria to trimethylamine (TMA), which is metabolized in the liver to TMA N-oxide (TMAO). TMAO impacts the liver through modulating glucose and lipid homeostasis and triggering adipose tissue inflammation [125]. TMAO also triggers platelet dysfunction, and circulating TMAO levels are associated with the severity of NAFLD and atherosclerosis, ventricular arrhythmias, and clinical outcomes [126,127,128,129,130]. It should be noted that TMAO may be regarded a crucial microbiota-derived biomarker of CVD and potentially also in the cross-talk between NAFLD and CVD; however, TMA/TMAO metabolism not only relies on an imbalance of the intestinal microbiota composition but also on dietary factors, co-metabolism, and host genetics [131].

Although suggested in several studies, the intestinal microbiota’s role in inducing inflammation and atherogenesis is incompletely understood. A recently published study demonstrated that introducing pro-inflammatory gut microbiota into mice enhances systemic inflammation and accelerates atherosclerosis [132]. In this context, it is intriguing that statins, independently of their effect in lowering low-density lipoprotein (LDL), may contribute to clinical benefits by their anti-inflammatory actions. In addition, it has recently been demonstrated that the use of statins is associated with a lower prevalence of gut microbiota dysbiosis, thereby potentially attenuating systemic inflammation [133].

Significant alterations in the intestinal microbiota were found in patients with coronary heart disease (CHD) complicated by NAFLD, showing a decrease in *Colinsella* and *Parabacterioides* as a potential explanation for the worse clinical outcome and disease progression compared to CHD without NAFLD [134].

It seems plausible that the gastrointestinal tract may be regarded as a site of origin of systemic inflammatory changes, thereby playing a crucial role in metabolic diseases such as NAFLD and CVD. Targeting metabolic inflammation may not only be attractive in combating atherosclerosis but may also become a central component in the management of other metabolic disorders in the future [87]. Although in its infancy, future studies focusing on causal interactions between inhabitants of the gut, including phages, viruses, and fungi, and metabolism may allow the development of new therapeutic agents for the treatment of NAFLD and CVD.

### 3.3. Dyslipidemia and Adipose Tissue (Dys-)Function

The mechanisms associating CVD with NAFLD are complex. It is the main challenge to identify where a distinct contribution of NAFLD occurs independently from the cardiovascular risk environment already constituted by MetS. However, several lines of evidence suggest that an affected liver may have an additional role in promoting CVD. From one aspect, this pertains to the dyslipidemia of NAFLD. Dyslipidemia is well established as closely linked to CVD, and low-density lipoprotein (LDL) cholesterol is accepted as a key driver of atherosclerosis [135]. The typical dyslipidemia of NAFLD is characterized by low high-density lipoprotein (HDL) cholesterol and high triglycerides, with moderately increased LDL cholesterol [136]. Lipid homeostasis is affected on several levels in NAFLD. While the liver is the central organ of lipid turnover, it does not store lipids in a healthy state [137]. In NAFLD, excess hepatic lipids derive from adipose tissue lipolysis with increased fatty acid flux to the liver, increased dietary uptake, increased de novo lipogenesis, and impaired export of newly synthesized VLDL (very low density lipoprotein) particles [138]. While typically in early stages, an increase in mitochondrial oxidation compensates, at least partially, for the excess in hepatocellular lipids [139], this becomes impaired at later stages. Normally, large VLDL particles lose their triglyceride moiety once secreted into circulation via lipoprotein lipase activity, turning into an equal number of atherogenic LDL particles with a dominant cholesterol content. LDL particles are taken up into the liver via the LDL receptor pathway [137]. The overproduction of VLDL particles in NAFLD triggers a series of lipoprotein abnormalities which result in the characteristic atherogenic dyslipidemia pattern that includes a predominance of particularly atherogenic, small dense LDL particles [140,141]. The severity of these lipid abnormalities appears to increase with more severe and advanced stages of liver disease [142,143]. These small dense LDL particles have a particular propensity to penetrate the vascular endothelium into the subendothelial space, which serves as an initiating event of atherosclerotic plaque formation. LDL cholesterol in the vascular wall is further subjected to oxidation and elicits an innate immune response via Toll-like receptors (TLRs) [144].

Atherosclerosis development is mainly mediated by apo-B-containing particles. Thus, besides LDL cholesterol-mediated risk for atherosclerosis, triglyceride-rich lipoproteins (TRLs) such as VLDL and IDL (intermediate density lipoprotein) also contribute to increased risk for CVD [145]. These particles also contain apo-C3, which likely has a role in activating TLRs and, therefore, the inflammasome reaction [146,147]. This, in turn, activates interleukin (IL)-1β family via caspase-1, inducing the prototypical pro-inflammatory mediators IL-1, IL-6, and CRP in the vascular wall [148]. This constitutes a link between lipid deposition and propagated inflammation in the vascular wall as the mechanism to CVD. This pathway may further be activated by saturated fatty acids, which are particularly increased in de novo lipogenesis of the steatotic liver and have been associated with increased cardiovascular risk such as palmitic acid C16:0 [149].

Besides its impact on lipids as a risk factor for CVD, NAFLD, like CVD, is an inflammatory disease, and pro-inflammatory cytokines such as TNF-α, interleukin-1β, or high-sensitive C-reactive protein (hs-CRP) are elevated in MetS-associated diseases [150]. This inflammatory milieu reflects pro-inflammatory mechanisms which are active in adipose tissue, the vascular wall, circulating immune competent cells, and also the liver. The liver is the physiological source for most acute-phase reactants, orchestrating the extent and duration of any immune response. In NAFLD, the liver becomes an additional permanent autonomous source of pro-inflammatory and pro-coagulant mediators [151,152,153]. Although the specific contribution of these additional NAFLD-derived factors to cardiovascular endpoints has not been demonstrated conclusively, from a pathophysiological point of view, it is plausible to consider the steatotic and inflamed liver as an enhancer of the inflammatory reaction in the vascular wall and as a catalyst of a prothrombotic coagulation state, favoring thrombus formation.

The gene polymorphisms of adiponectin rs266729 [154], adiponectin-encoding gene (ADIPOQ), leptin receptor (LEPR), apolipoprotein C3 (APOC3), peroxisome proliferator-activated receptors (PPAR), sterol regulatory element-binding proteins (SREBP), transmembrane 6 superfamily member 2 (TM6SF2), microsomal triglyceride transfer protein (MTTP), tumor necrosis factor-alpha (TNF-α), and manganese superoxide dismutase (MnSOD) have been reported to be related to NAFLD and coronary artery disease [155].

### 3.4. Endothelial Dysfunction

Endothelial dysfunction precedes atherosclerosis. In NAFLD, elevated levels of asymmetric dimethyl arginine, an endogenous antagonist of nitric oxide synthase, are observed [156,157]. Increased serum homocysteine levels are seen in NAFLD, primarily due to changes in methionine metabolism, which changes the production and catabolism of homocysteine in the liver. Hyperhomocysteinemia is associated with increased intrahepatic vascular resistance, which impairs nitric oxide formation, and increased oxidative stress activates platelets. Furthermore, systemic inflammation (Section 3.1) increases endothelial dysfunction, alters vascular tone, and enhances vascular plaque formation. These mechanisms are supported by the clinical findings in a study of NAFLD patients that found significantly reduced flow-mediated vasodilation compared with age- and sex-matched control subjects [158]. As endothelial dysfunction is the main mechanism of erectile dysfunction in men, erectile dysfunction was shown to be a relevant issue in patients with NAFLD [159].

## 4. Current and Future Treatment Options for NAFLD Complicated by CVD

**Figure 3:**
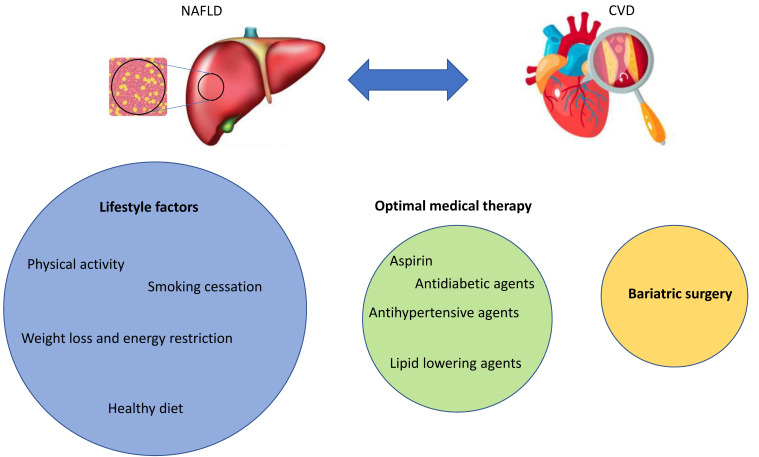
Besides lifestyle intervention and, for some patients, surgery, a number of established and emerging CV medications might play a role in the treatment of NAFLD complicated by CVD.

Besides lifestyle intervention and, for some patients, surgery, a number of established and emerging CV medications might play a role in the treatment of NAFLD complicated by CVD (Figure 3).

### 4.1. Lifestyle Interventions

There are currently no Food and Drug Administration (FDA)-approved pharmacological therapies for NAFLD, and lifestyle interventions including smoking cessation, weight loss, healthy nutrition, and exercise remain the cornerstones for treatment [160].

#### 4.1.1. Smoking Cessation

There is associative evidence available that smoking contributes to the development of NAFLD via direct and indirect mechanisms [161]. As smoking is an established cardiovascular risk factor and smoking cessation is central to the prevention of cardiovascular events, also in subjects with NAFLD, smoking cessation is desirable.

#### 4.1.2. Weight Loss and Energy Restriction

Energy restriction induces weight loss and the reduction of liver fat, independent of the macronutrient composition of the diet [162]. Consequently, a reduction of 500–1000 kcal of daily energy intake might induce a weight loss of 500–1000 g/week. A total of 7–10% weight loss is desirable in overweight and obese patients with NAFLD, as weight loss leads to significant reduction in hepatic steatosis [163]. Cognitive–behavioral treatment, a combination of exercise with energy restriction, and long-term maintenance approaches delay vascular and metabolic complications in NAFLD and the onset of T2DM [164].

#### 4.1.3. Healthy Nutrition

Although the main aspect in healthy nutrition is energy restriction, irrespective of macronutrient composition, low-to-moderate fat and moderate-to-high carbohydrate, low-carbohydrate ketogenic, or high-protein diets have been suggested by some studies to be beneficial beyond the effect of sole energy restriction. As evidence on macronutrient composition and the best diet to prevent or treat NAFLD is scarce and conflicting, energy restriction should be the main aspect to focus on in evidence-based nutrition counselling [160]. Guidelines for both the treatment of NAFLD and cardiovascular disease prevention currently recommend adaption of the macronutrient composition to the pattern of the Mediterranean diet. This, in brief, involves a vegetable-based diet with plant oils (olive oil) as the main dietary fat source, whole grains as the main dietary source of carbohydrates, and predominant consumption of plant protein such as nuts and legumes with avoidance of processed red meat and limited amounts of dairy products and fish or white meat. From a cardiovascular perspective, the additional restriction of salt consumption (DASH diet, dietary approach to stop hypertension) complements the dietary pattern currently considered healthy. Avoiding fructose-containing beverages and foods, however, has been shown to be associated with lower prevalence of NAFLD and appears to be helpful for CV risk reduction [165]. Alcohol intake should be moderated and limited to 30 g in men and 20 g in women, as alcohol intake below this “risk threshold” was associated with a lower prevalence of NAFLD, NASH, and even lower fibrosis at histology [166,167,168]. However, absolute alcohol abstinence is mandatory in advanced fibrosis and NASH-cirrhosis to reduce the risk of worsening liver dysfunction and HCC [169]. Coffee was shown to be protective in NAFLD, as in liver disease of other etiologies, reducing histological severity and liver-related outcomes [170].

#### 4.1.4. Exercise

Exercise and physical activity are universally recommended for the promotion of metabolic and vascular health, i.e., in healthy or at-risk subjects and patients [171]. Hence, moderate-intensity aerobic physical activities in 3–5 sessions (a total of 150–300 min/week) are advised. Resistance training in two sessions per week has additional effects and promotes musculoskeletal fitness, with effects on metabolic risk factors, mainly linked to augmented muscular glucose utilization. A sedentary lifestyle induces fatigue and daytime sleepiness and reduces compliance with exercise recommendations. Physical activity is dose-dependent and vigorous (running) rather than moderate exercise (brisk walking) carries the full benefit, including for NASH and fibrosis [172,173]. Any engagement in physical activity or increase over previous levels is better than continuing inactivity. Long-term adherence is a major limitation for exercise and physical activity to unfold its full potential [174].

### 4.2. Medication

#### 4.2.1. Aspirin

Acetylsalicylic acid (ASA) still constitutes a cornerstone in antiplatelet therapy once a definitive diagnosis of atherosclerotic disease is established [10,11]. There are data from basic and small observational studies suggesting an effect of ASA on the progression of NAFLD [175]. The intake of ASA could be associated with a reduction in liver fibrosis, and several observational studies showed a lower incidence of liver fibrosis and evidence of slower fibrosis progression in patients taking ASA [176,177]. However, these data remain speculative, and there is no randomized evidence supporting the use of ASA in patients with NAFLD without manifestation of atherosclerosis [178]. On the other hand, the safety profile of ASA is good even in patients with manifestation of liver cirrhosis without esophageal varices [179]. ASA should, therefore, be administered in NAFLD patients based on the recommendations aiming at the prevention of cardiovascular events [10,11]. From a mechanistic perspective, ASA intake via reduced platelet activation and sinusoidal microthrombus formation could limit local hypoxia and, thus, fibrogenesis in a similar fashion to the action of low-molecular-weight heparin [180,181].

#### 4.2.2. Lipid-Lowering Agents

Lipid-lowering drugs, among which statins are the most widely prescribed and potent substances with the best evidence, are an essential component in the drug modification of cardiovascular risk [10,11,182].

Although statins have hepatic toxicity as a rare side effect, statins should not be withheld from patients with NAFLD for primary and secondary prevention of cardiovascular events [183]. This is of high clinical relevance as statins should also be administered to patients with elevated transaminases (due to their NAFLD), but this is often not practiced in daily reality. In a study that analyzed 261 patients with NAFLD, only 13% received statin therapy, which may be considered too low given the high coincidence of NAFLD, CVD, and metabolic syndrome [184,185]. In both the GREACE study and the IDEAL study, atorvastatin was shown to reduce risk compared to a less potent lipid-lowering therapy with adequate safety, with a particular cardiovascular benefit for subjects with elevated transaminases [186,187,188]. Furthermore, in several studies, surrogate parameters for a reduction in the progression of the liver disease NAFLD could be detected in addition to a cardiovascular risk reduction [189,190]. Therefore, statin therapy is not contraindicated in patients with NAFLD but is likely particularly beneficial from both a cardiological and a hepatological perspective.

Similarly, the use of ezetimibe, another lipid-lowering agent supported by guidelines, is considered to be safe and effective for the prevention of cardiovascular events in NAFLD [191]. However, the use of ezetimibe in NAFLD patients beyond the indication supported by the lipid-lowering evidence is currently speculative, as is a possible effect of ezetimibe on liver-related endpoints [10,11,182,191].

The PCSK9 inhibitor (PCSK9i) represents a relatively new substance class in lipid-lowering therapy which has an excellent potency to lower LDL cholesterol especially [192,193,194]. PCSK9i has a relatively narrow indication in cardiological patients with very high cardiovascular risk, statin intolerance, or persistently high LDL concentrations despite statin therapy [195]. Basic science and clinical studies suggest that high intrahepatic or circulating levels of PCSK9 play an important role in muscle and liver lipid storage and may contribute significantly to the pathogenesis and progression of NAFLD [196]. Conversely, PCSK9 inhibition increases LDL via the LDL receptor-mediated pathway and, therefore, lipid content of hepatocytes. Based on these data, it is speculative whether PCSK9i has an impact on the cardiovascular and hepatological complications of NAFLD, and several indirect studies evaluating, e.g., berberine suggest a beneficial effect [197,198]. No liver-related signals were observed in large PCSK9 Fourier and Odyssey outcome studies, and thus, it appears to be safe in the target population with regard to liver disease. However, since randomized outcome studies in NAFLD patients investigating the efficacy and safety of PCSK9i are still pending, this assumption remains speculative [196]. Therefore, based on the available evidence, NAFLD diagnosis should not restrict PCSK9i therapy when indicated for primary or secondary prevention.

#### 4.2.3. Antihypertensive Agents

Among NAFLD patients, the prevalence of arterial hypertension varies from 40% to 70%, and evidence from prospective studies from France and Germany has shown that NAFLD is associated with a 2–3-fold increase in the incidence of arterial hypertension over time [199,200]. In a population-based cohort of hypertensive (*n* = 433) and normotensive (*n* = 457) subjects from Finland, fatty liver was associated with significantly higher ambulatory daytime and nighttime systolic blood pressure levels [201].

Furthermore, the severity of progressive NASH tends to increase in patients with hypertension, although this association does not appear to be as strong as that with T2DM.

Despite the strong association between NAFLD and arterial hypertension, sufficient evidence on the comparative benefit of certain antihypertensive agents in NASH is lacking. Thus, antihypertensive management in patients with NAFLD is routinely performed along with current guidelines irrespective of this diagnosis, although several pharmaceutical agents harbor theoretical advantages over others. Betablockers are highly effective at lowering systolic and diastolic blood pressure but may also adversely affect physical activity in some patients with NAFLD and, thus, counteract calorie expenditure. Furthermore, betablockers suppress stress reactions to hypoglycemia in NAFLD patients concomitantly treated for T2DM. For these reasons, angiotensin-converting enzyme inhibitors (ACE-I) and angiotensin receptor blockers (ARB) are favored groups of agents. Another reason is their nephroprotective potential, bearing in mind the high prevalence of T2DM and consecutive kidney dysfunction among NAFLD patients [202]. Additionally, both groups of agents target myofibroblasts and hepatic stellate cells in experimental models of liver fibrosis and counteract portal hypertension [203], but current evidence suggests exercising caution in the extended use of ACE-I/ARB solely for the purpose of managing fibrosis in NAFLD patients [204]. Once cirrhosis with significant portal hypertension or signs of hepatic dysfunction evolve, treatment with both ACE-I and ARB should be reevaluated and a risk–benefit analysis should be performed due to a higher incidence of complications, including kidney failure and sodium and potassium derangements.

#### 4.2.4. Antidiabetic Agents

The relevance of T2DM in patients with NAFLD is twofold: First, over half of all NAFLD patients reveal T2DM, and second, the presence of T2DM promotes the progression of NAFLD and is a predictor of advanced fibrosis and mortality [205]. T2DM is not only associated with NAFLD but is also a significant risk factor of progressive liver disease per se. Based on data from a recent meta-analysis including 22.8 million individuals followed up for a median of 10 years, T2DM is associated with an increased risk of incident severe liver disease events (adjusted HR (hazard ratio) 2.25, 95% CI 1.83–2.76, *p* < 0.001) [206]. Conversely, patients with NAFLD have an up to twofold increased risk of subsequent incident T2DM [202]. Therefore, patients with NAFLD should be screened and surveilled for T2DM and, vice versa, diabetic patients should be assessed for possible NAFLD.

Management of T2DM in NASH patients must take into account coexisting comorbidities and consider its impact on underlying cofactors of NAFLD. This is particularly true for overweight. Metformin is the first choice of treatment for T2DM and also in NAFLD [160], although the evidence for an improvement in histological changes such as liver fat or fibrosis is weak. Euglycemic control with metformin is satisfactory in many patients at relatively low costs, and metformin causes no weight gain. Moreover, some retrospective human data point to an anti-tumor effect of metformin on HCC [207], which may also support its use in NAFLD, a disease with a substantial risk of HCC also in non-cirrhotic patients.

In patients who do not achieve adequate glycemic control with metformin, therapeutic escalation can be undertaken with the following agents:

1. Thiazolidinediones (TZDs): TZDs are selective and potent PPARγ agonists by which they act as insulin sensitizers and, thus, synergistically with circulating insulin. Through this effect, TZD overcomes insulin resistance as a hallmark of NAFLD. Of the three compounds introduced to the market, only pioglitazone currently remains available after troglitazone was withdrawn due to severe hepatotoxicity and rosiglitazone was linked to adverse cardiovascular incidents. Pioglitazone was compared to vitamin E and a placebo in the PIVENS trial and given for 96 months at a dose of 30 mg daily. Elevated serum liver enzymes were reduced with both agents, and both were associated with significant reductions in hepatic steatosis (*p* = 0.005 for vitamin E and *p* < 0.001 for pioglitazone) and lobular inflammation (*p* = 0.02 for vitamin E and *p* = 0.004 for pioglitazone). However, improvements in fibrosis scores were observed for neither active treatment. For the latter, however, a meta-analysis of five randomized controlled trials with pioglitazone in NAFLD patients [208] found an association with improved advanced fibrosis (OR (odds ratio), 3.15; 95% CI, 1.25–7.93; *p* = 0.01), fibrosis of any stage (OR, 1.66; 95% CI, 1.12–2.47; *p* = 0.01), and NASH resolution (OR, 3.22; 95% CI, 2.17–4.79; *p* < 0.001), even when given to subjects without diabetes [209]. Relevant disadvantages of pioglitazone in the treatment of T2DM in NAFLD patients include significant weight gain, fluid retention, and some evidence from observational studies demonstrating an increased risk of bladder cancer [210]. Pioglitazone is the current treatment of choice for NAFLD in subjects with T2DM.
2. Sodium-glucose cotransporter-2 (SGLT-2) inhibitors: SGLT2 inhibitors are compounds that inhibit SGLT-2 proteins located in the renal tubules of the kidneys, which are responsible for the reabsorption of glucose, thereby increasing the loss of glucose via urinary excretion. There are three SGLT-2-selective inhibitors approved by the Food and Drug Administration (FDA), namely canagliflozin, dapagliflozin, and empagliflozin. In a recent systematic review of studies investigating SGLT-2 inhibitors in NAFLD patients, seven studies were reviewed, of which six demonstrated certain improvements by SGLT-2 inhibitors in NAFLD. Meanwhile, five studies showed a reduction in steatosis, and only one study suggested an improvement of NASH histology features [211]. Beyond these liver-related endpoints, SGLT-2 inhibitors also favorably impact weight, cardiovascular, and nephrological outcomes, rendering them a satisfactory and increasingly popular, albeit not ideal, group of agents to treat T2DM in NAFLD. Urinary tract infections limit their use in a relevant number of patients [212].
3. Glucagon-like peptide 1 (GLP-1) agonists. GLP-1 is an antihyperglycemic hormone that induces pancreatic β cells to secrete insulin, and it was demonstrated that patients with NAFLD reveal insufficient GLP-1 secretion [213]. Thus, pharmaceuticals that offset this imbalance to improve glucose homeostasis are attractive candidates for treating T2DM in the context of NAFLD. Indeed, GLP-1 agonists have recently gained much attention based on clinical data from trials that provide strong evidence for a powerful effect of GLP-1 agonism on hepatic endpoints in patients with NAFLD and also a reduction in CV endpoints.

In the LEAN study, 26 patients with biopsy-proven NASH were randomly assigned to receive liraglutide at 1.8 mg daily and 26 were assigned to a placebo as part of a multicenter, double-blinded, randomized, placebo-controlled, 48-week phase 2 trial [214]. Premier findings were an improvement of NASH histology in 39% of NASH patients on liraglutide compared to only 9% of NASH patients receiving the placebo who revealed improved histology. In addition, worsening of fibrosis occurred in 36% of patients on the placebo but only 9% in those receiving liraglutide. Tolerability was acceptable overall, with the expected gastrointestinal side-effects from liraglutide including diarrhea, constipation, loss of appetite, and nausea. It can be assumed that some of the improvement in liver-related endpoints can be ascribed to weight loss in a high proportion of patients. Similar observations were recently made with semaglutide, another GLP-1 agonist available for both subcutaneous injection and orally, which caused a significant drop in elevated serum liver enzyme levels and markers of inflammation in patients treated for T2DM and/or overweight [215]. Comparable observations have also been recorded for lixisenatide [216] and dulaglutide [217].

Importantly, with regard to NAFLD patients with cardiovascular comorbidities, GLP-1 agonists have been shown to reduce cardiovascular and all-cause mortality and reduce heart failure and the progression of chronic kidney disease (CKD).

Overall, GLP-1 agonists appear to be the most pertinent group of therapeutic agents since they have the potential to reduce body weight as a crucial clinical condition in patients with NAFLD, and the availability of an orally administered compound can be considered a true turning point in the treatment of NAFLD. Their specific use to treat NAFLD, however, is not licensed, and additional studies with liver-specific primary endpoints are required to decipher their actual value in the treatment of NAFLD.

Other antidiabetic drugs including insulin, sulfonylurea agents, and dipeptidyl peptidase IV inhibitors are either less studied in patients with NAFLD, reveal significant disadvantages (weight gain and worsening of sarcopenia), or show no benefit for the underlying liver disease. Their use is therefore either discouraged (insulin) or, at best, a second-line option, should more favorable compounds fail or prove intolerant.

Furthermore, currently ongoing NASH trials are frequently focused on liver-specific outcomes only, and the effect of CV outcomes will need to be studied further, particularly in light of available data of SGLT2 inhibitors and GLP-1 agonists which appear to improve both. Additionally, it will be interesting to see whether treatment of liver disease via liver targets will have an impact on cardiovascular disease outcomes as this may provide important insights into the causality relation of liver and vascular disease in the future. A practical and comprehensive overview on the use of antidiabetic drugs in NAFLD is provided elsewhere [218].

### 4.3. Surgery

If lifestyle intervention and adequate treatment of comorbidities are not sufficient to reduce the overall risk, bariatric surgery in NAFLD is a final option. Bariatric surgery may induce weight loss and improve the detrimental effects of MetS and T2DM. Recent data demonstrated significant effects of bariatric surgery on GLP-1 and other gut hormones and important beneficial changes in lipid, metabolic, and inflammatory abnormalities in NAFLD. Bariatric surgery may, therefore, reverse some of the pathological liver changes in NAFLD and NASH patients and reduce CV outcomes [219].

## 5. Conclusions

In conclusion, overwhelming evidence suggests an association of CVD with NAFLD; however, the related mechanisms remain largely speculative. It is, however, likely that identical mechanisms contribute to the development of CVD and NAFLD, with lifestyle factors such as smoking, sedentary lifestyle with poor nutrition habits, and physical inactivity being major aspects. These behavioral factors, among others, trigger changes in the gut microbiota, inflammation, dyslipidemia, and oxidative stress, leading to metabolic syndrome, diabetes, and obesity as well as atherosclerosis. Treatment options to counteract both the progression and development of CVD and NAFLD include lifestyle interventions, optimal medical therapy of comorbid conditions, and, as final possibility, bariatric surgery (Figure 3). As no causal pharmacotherapy of NAFLD is available, further research is urgently needed to address the unmet needs of a growing population with NAFLD and CVD.

## Data Availability

No new data were created or analyzed in this study. Data sharing is not applicable to this article.
